# Ballistic protective properties of material representative of English civil war buff-coats and clothing

**DOI:** 10.1007/s00414-020-02378-x

**Published:** 2020-07-21

**Authors:** Brian May, Richard Critchley, Debra Carr, Alan Peare, Keith Dowen

**Affiliations:** 1 Centre for Defence Engineering, Cranfield University, Defence Academy of the United Kingdom, Shrivenham, SN6 8LA UK; 2 Defence and Security Accelerator, Porton Down, Wiltshire, SP4 0JQ UK; 3 Royal Armouries, Armouries Drive, Leeds, LS10 1LT UK

**Keywords:** Leather, Linen, Wool, Behind armour blunt trauma, Pencilling, 12-bore

## Abstract

One type of clothing system used in the English Civil War, more common amongst cavalrymen than infantrymen, was the linen shirt, wool waistcoat and buff-coat. Ballistic testing was conducted to estimate the velocity at which 50% of 12-bore lead spherical projectiles (*V*_50_) would be expected to perforate this clothing system when mounted on gelatine (a tissue simulant used in wound ballistic studies). An estimated six-shot *V*_50_ for the clothing system was calculated as 102 m/s. The distance at which the projectile would have decelerated from the muzzle of the weapon to this velocity in free flight was triple the recognised effective range of weapons of the era suggesting that the clothing system would provide limited protection for the wearer. The estimated *V*_50_ was also compared with recorded bounce-and-roll data; this suggested that the clothing system could provide some protection to the wearer from ricochets. Finally, potential wounding behind the clothing system was investigated; the results compared favourably with seventeenth century medical writings.

## Introduction

At the end of the seventeenth century English Civil Wars, many survivors of Alexander Popham’s Parliamentary Forces marched home to Littlecote (Wiltshire, UK), laid aside their weapons and armour and returned to their peacetime occupations [3]. Considered to be the last surviving Civil War armoury in Britain, the Littlecote House collection appears to have largely been assembled by Alexander Popham in the mid-seventeenth century [24] and was acquired by The Royal Armouries in 1985 [23]. Amongst the collection were 36 buff-coats [3] which form the largest single surviving group of such items in the world [23].

Buff-coats have been described as ‘an oil-tanned, leather garment, typically with thigh to knee-length skirts used in place of, or in conjunction with, plate armour’ [11]. Oil-tanned refers to the buff leather production technique which was based upon the oxidation of marine animal or fish oils [13]. Although buffalo hide may have given the garment its name, cattle and deer hides were more commonly used [11]. In the seventeenth century, buff-coats were one of the most widely worn forms of body-protection amongst the cavalry of many European nations [11].

Analysis of the Littlecote collection buff-coats suggested that they were individually tailored for the men wearing them [20]. Despite their relatively widespread use amongst the cavalry, use as a protective garment during the English Civil Wars, their effectiveness as a protective garment is not known [11]. Buff-coats were commonly worn over civilian clothing (linen shirt and wool waistcoat) during the English Civil War as the use of uniforms was not common [21].

The musketeers of the English Civil War typically carried a matchlock musket, with the 12-bore musket being the most common calibre [15]. The projectiles fired using muskets were typically lead spheres and were accelerated by burning gunpowder (black powder) within the barrel of the weapon and behind the projectile. Black powder is a pyrotechnic mixture containing a fuel (charcoal and sulphur) and an oxidizer (potassium nitrate) [1]. The rapid burning of black powder produces large quantities of gases that create a high pressure within the confined space of the barrel accelerating the projectile along and out of the barrel.

The Royal Armouries requested that the ballistic protective properties of a one type of clothing system (linen shirt, wool waistcoat and buff-coat) worn during the English Civil War be estimated and that the behind clothing wounding be considered to add to the international literature on this topic.

## Materials and method

### Leather, wool and linen

In the current work, oil-tanned leather (Clayton of Chesterfield, UK), plain-woven wool fabric (Historical Management Associates Ltd., Bristol, UK) and plain-woven linen fabric (The Tudor Tailor, Nottingham, UK) were used to represent buff-coat, wool waistcoat and linen shirt clothing layers. The linen was washed before use according to ISO 6330:2001 and flat dried to Procedure C [4]. The thicknesses of the leather, wool and linen were measured using a Mitutoyo Thickness Gauge Model ID-C1012MB with a tolerance of ± 0.02 mm. Leather thickness was determined according to ISO 2589:2016 [7] whilst density was calculated according to ISO 2420:2017 [6]. Wool and linen fabric mass per unit area was calculated according to BS 2471:2005 [5]. Masses of specimens were measured using an Oxford A2204 scale with a tolerance of ± 1 mg. Specimens for testing were cut 250 × 250 mm.

### Gelatine

Gelatine blocks (10% by mass and conditioned to 4 °C) are commonly used as a tissue simulant in wound ballistic experiments [10]. Mabbott [16] suggested that 10% (by mass) gelatine conditioned to 4 °C closely replicates the thorax when considering penetrations for specific projectiles. Six gelatine blocks (250 × 250 × 500 mm) were made using Gelita® Ballistic 3 gelatine (Lot 073650) in aluminium containers and conditioned for 36 h at 4 °C [16]. The blocks were calibrated prior to ballistic testing by firing a 5.5-mm diameter steel ball bearing (Atlas Ball & Bearing Company, Walsall, Batch Number 13103003) in to the top right-hand corner of each block using a gas-gun (‘Gas-gun’ section). Ballistic impacts were recorded using a Phantom V1212 high-speed camera (12,000 frames per second). Impact velocity of the ball bearing was calculated from the high-speed video of each impact and along with the depth of penetration (DoP) into the block compared with prior calibration data [16]. After calibration, each block was cut in half (250 × 250 × 250 mm) for use in the ballistic testing.

### Projectiles

**Fig. 1:**
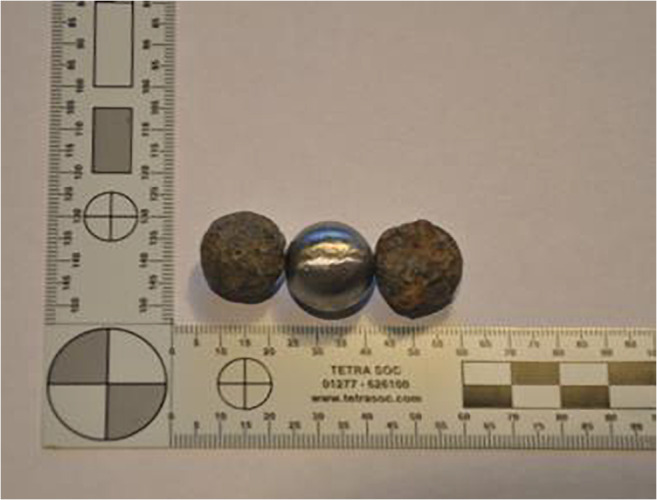
Modern cast test projectile between two historical examples recovered from the Thames Foreshore

Firth and Dowen suggested that 12-bore musket balls were the most common projectile used during the English Civil War [12, 14], and this calibre was selected as a representative projectile for the current work. Twelve lead projectiles were cast using a spherical 12-bore musket ball split mould (Fig. 1). Projectile diameter, mass, elemental analysis (Hitachi SU3500 Scanning Electron Microscope fitted with an Ametek Octane Plus Microscope and using TEAM (Texture & Elemental Analytical Microscopy)) and Vickers hardness (Indentec Hardness Tester, Model HWDM-7) were measured and compared with data for historical specimens provided by one of the authors (KD).

### Gas-gun

**Fig. 2:**
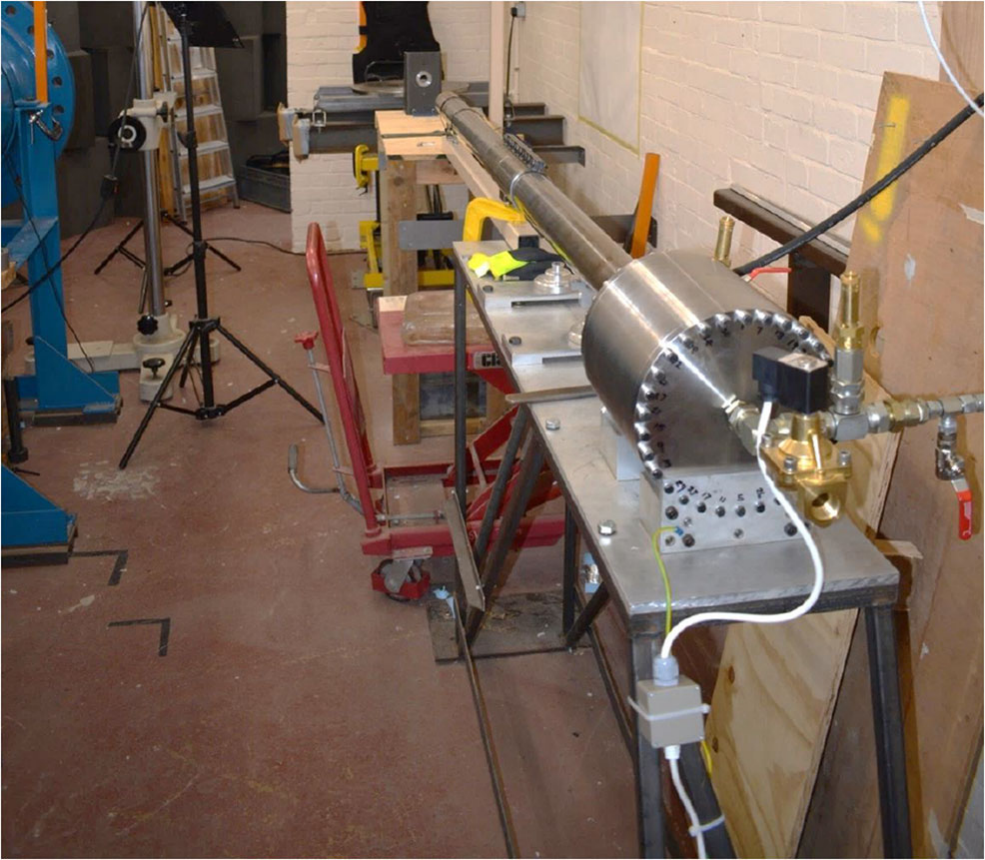
Shrivenham gas-gun

Projectiles (ball bearings and lead spheres) were fired in purpose designed sabots using a gas-gun system (Fig. 2). Projectile velocity was adjusted by altering the nitrogen pressure used to operate the gas-gun (Annex). To ensure that only the projectile impacted the target, a sabot stripper was placed 820 mm from the end of the gas-gun barrel.

### Ballistic testing

**Fig. 3:**
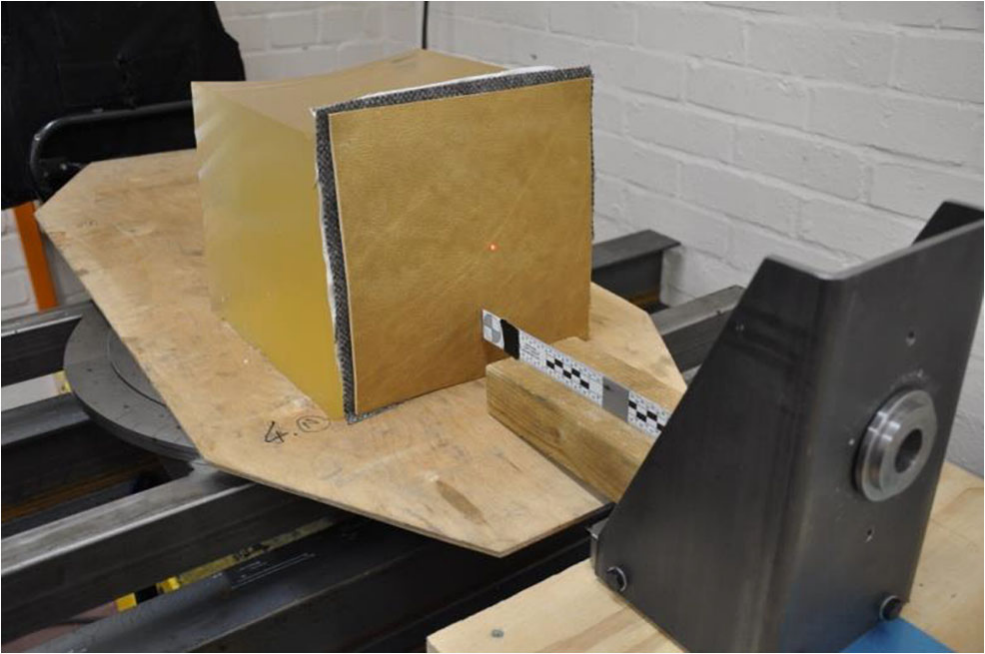
Configuration of target materials

Clothing layer specimens (leather, wool fabric, linen) were mounted on the anterior surface of a block of gelatine using dress-makers pins in each corner (Fig. 3). The leather formed the impact face of the resulting target which was impacted centrally with a single 12-bore lead sphere at varying velocities. Each impact was recorded using a high-speed video as described in the ‘Gelatine’ section. The ballistic performance of the clothing system, estimated *V*_50_, was calculated with reference to AEP-2920(A) [19]. Twelve shots were fired in total and a single six-shot *V*_50_ was calculated from the three fastest non-perforating impacts and the three slowest perforating impacts with a ≤ 40 m/s spread across all velocities [19]. Ideally multiple *V*_50_ data would be obtained and the *V*_50_ expressed as a mean of these results with an associated standard deviation. However, in this work, the amount of material representative of the clothing system was limited and therefore the *V*_50_ calculated should be considered indicative of the protective performance of the clothing system. The distance at which the projectile would decelerate from the muzzle velocity to the estimated *V*_50_ was calculated and compared with known effective engagement distances. Previously published bounce-and-roll data for 12-bore lead spheres [18] was also considered so that the protective capabilities from ricochet could be considered. Finally, probable wounding effects were considered by examination of the high-speed video and post-testing dissection of the gelatine blocks.

## Results and discussion

### Clothing layers

The physical properties of the clothing layers are given in Table 1. As expected, the leather was the thickest and heaviest, and the linen was the thinnest and lightest. The variability of the leather was smaller than the other two layers due to the nature of the processing of this material (i.e. trimmed). The thickness of the leather compared favourably with thickness data for buff-coats from The Royal Armouries Collection which varied from 1.52 to 5.33 mm (mean = 3.56 mm) [23].

**Table 1:** Physical properties of clothing layers

Clothing layer	Thickness (mm)		Leather density (g/m^3^)/fabric mass per unit area (g/m^2^)	
	Mean	SD	Mean	SD
Leather	3.36	0.28	0.98	0.07
Linen	0.38	0.04	182.62	2.12
Wool	2.01	0.04	367.19	2.22

### Lead spherical projectiles

The cast lead spheres had a mean diameter of 18.55 mm (SD = 0.12 mm), a mean mass of 36.1 g (SD = 0.18 g) and a mean Vickers hardness of 24.6 Vickers (SD = 0.69 Vickers). The modern projectiles were a lead/antimony alloy. These properties can be compared with data for a historical example provided by KD. The historical projectile had a mass of 37.2 g, so slightly heavier than the modern spheres. No measurement of the outside diameter of the historical projectile was undertaken due to the formation of a corrosion layer as the measured dimension would describe what the dimension of projectile plus corrosion is and not its original dimensions when cast.

The alloying of the lead with antimony resulted in a harder projectile; the historical projectile had a hardness of 5.4 Vickers. Some information regarding historical musket balls is also available in the literature and compares favourably with the modern spheres manufactured for the current work. For example, a musket ball from the Battle of Marston Moor had a hardness of 6.32 Vickers and was 99.7% lead; musket balls recovered from the Battle of Edgehill had a mean diameter of 18.51 mm and a mean mass of 37.9 g [26].

### Gelatine calibration

**Fig. 4:**
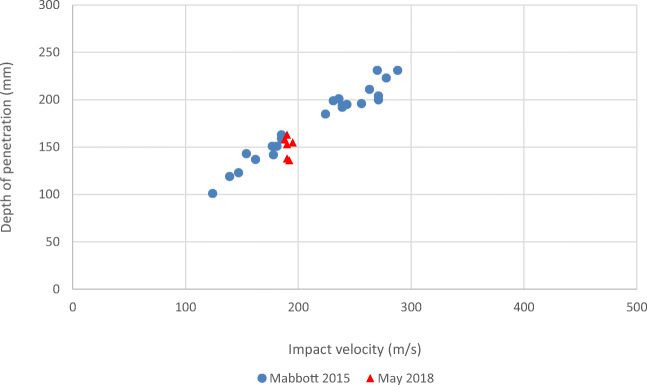
Calibration data compared with previous data

The mean impact velocity of the calibration shots was 190.8 m/s (SD = 2.4 m/s) and the mean DoP was 150.5 mm (SD = 11.0 mm). This data compared favourably with previous calibration data [16] providing confidence in the quality of the prepared gelatine blocks both within the batch used, but also when compared with previous data (Fig. 4).

### Ballistic testing

Using the ballistic test data (Table 2), a single six-shot 12-bore lead sphere estimated *V*_50_ of 102 m/s (SD = 7.5 m/s) was calculated. This suggests that in 50% of cases a 12-bore lead sphere impacting the target at a velocity of 102 m/s would perforate the target.

**Table 2:** Ballistic testing results

Shot	Non-perforation (m/s)	Perforation (m/s)
1	102	
2	111	
3		128
4		120
5		117
6		115
7		112
8		109
9		105
10		97
11	83	
12	91	

### Deceleration of a musket ball in air

**Fig. 5:**
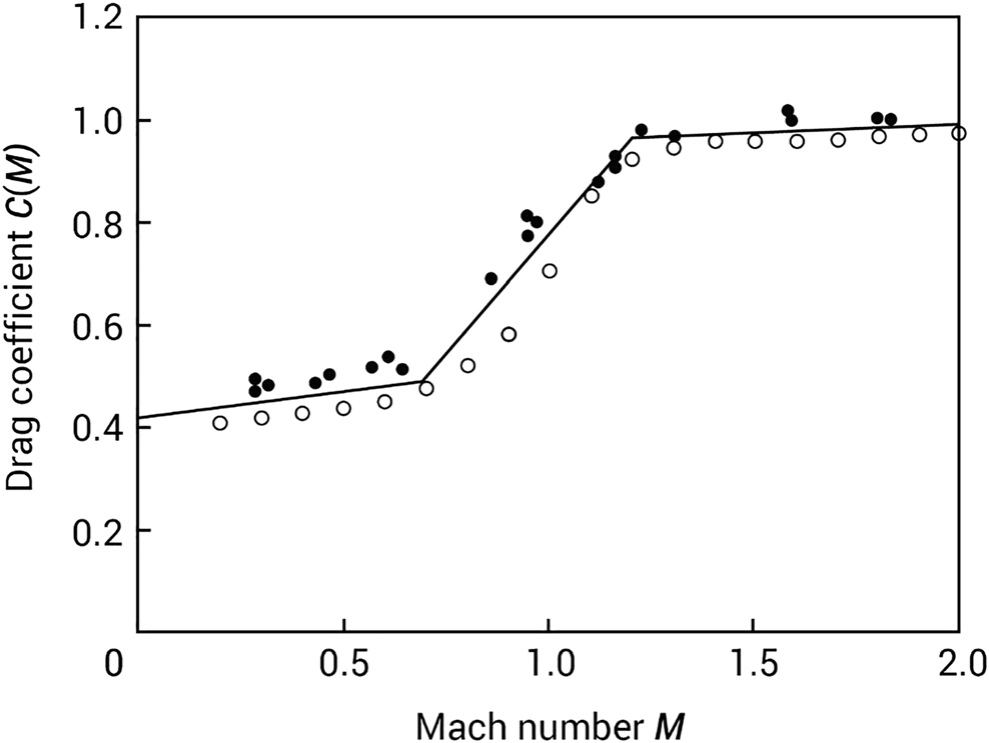
Mean drag coefficient versus Mach number for Reynolds number 10,000 (open points) and 9/16″ spheres (closed points). Adapted from [2]

Understanding the distance from the end of the weapon system’s muzzle that a musket ball would need to travel to have a velocity of 102 m/s would allow comment on the effectiveness of the clothing system at Civil War engagement distances. The muzzle velocity of a seventeenth century 12-bore musket has been estimated as 457 m/s [18]. Allen [2] published formulae which are valid between Mach 2 (681 m/s) and Mach 0.2 (68 m/s) to relate velocity to distance for spherical projectiles. The drag coefficient for a sphere changes as the projectile decelerates through the speed of sound (Fig. 5); therefore, three steps need to be considered (i) deceleration from 457 m/s to Mach 1.2 (408 m/s); (ii) Mach 1.2 to Mach 0.7 (238 m/s) and (iii) Mach 0.7 to 102 m/s [2].

To calculate the velocity at any distance between the firing point, when muzzle velocity is above Mach 1.2, and the distance at which the projectile decelerates to Mach 1.2 (*x*_1_):

$$ v=\frac{0.92{M}_0{v}_{\mathrm{s}}}{\left(0.92+0.0375{M}_0\right)\exp \left(\frac{0.69x}{k_{\mathrm{z}}}\right)-0.0375{M}_0} $$

where *v* is velocity of the projectile (m/s),

*v*_s_: is the velocity of sound (m/s),
*x*: is the distance that the projectile has travelled (m) and
*M*_0_: is the muzzle velocity of the projectile expressed as a Mach ratio.
*k*_z_: is a scaling factor with the same units as distance x (i.e. *m*) and is defined as:

$$ {k}_{\mathrm{z}}=\frac{D{\rho}_{\mathrm{p}}}{\rho_{\mathrm{a}}} $$

where *D* is the diameter of the projectile (18.5 mm),

*ρ*_p_: is the density of the projectile (10.9 g/cm^3^) and
*ρ*_a_: is the density of air at 20 °C (1.20 × 10^−3^ g/cm^3^).

Therefore, *k*_z_ is 168.04 m.

For a projectile with an initial velocity greater than Mach 1.2, the distance at which it will have decelerated to Mach 1.2 (*x*_1_) is obtained by using the following equation:

$$ {x}_1=1.44298{k}_{\mathrm{z}}\mathit{\ln}\left(\frac{0.80417{M}_0}{0.92+0.0375{M}_0}\right) $$

For a 12-bore musket ball with a muzzle velocity of 457 m/s (Mach 1.3), it will decelerate to Mach 1.2 (412.8 m/s) after a distance of 23.1 m.

To determine the distance at which it would decelerate to Mach 0.7 (*x*_2_), Allen used the following equation:

$$ {x}_2={x}_1+1.05173{k}_{\mathrm{z}} $$

Therefore, the distance from the firing position at which the 12-bore projectile would have decelerated to Mach 0.7 (240.8 m/s) is 199.9 m.

For the final phase deceleration profile:

$$ v=\frac{0.2926{v}_s}{0.495\mathit{\exp}\left(\frac{0.3135\left(x-{x}_2\right)}{k_{\mathrm{z}}}\right)-0.077} $$

The equation can be re-arranged to solve for a distance (*x*) given a known velocity.

$$ x=\frac{k_{\mathrm{z}}}{0.3135}\left\{\mathit{\ln}\left(\frac{\frac{0.2926{v}_s}{v}+0.077}{0.495}\right)\right\}+{x}_2 $$

Therefore, the 18.5-mm diameter musket ball will decelerate from 457 to 102.5 m/s in approximately 607 m. Typical engagement distances during the English Civil Wars were much less than this, e.g. at the 2nd Battle of Newbury, Royalist Forces were ordered not to give fire until they came within a pike’s length of the enemy (~ 5 m) [21]. Therefore, the clothing system would not have protected the wearer from a 12-bore musket ball impacting a person on a direct trajectory from the firing weapon at engagement distances of the period.

### Protection from ricochet

Reports from the Battle of Rathconnel discuss spent bullets (ricochets and bounces) hitting officers at close quarters with the enemy and failing to inflict penetrating injuries [21]. Clearly, the protective equipment worn was of some use against the battlefield threats of the day. Miller’s live-firing experiments recorded initial flight distances of between 153 and 203 m before impacting the ground for the first time [18]. In some tests, following impact with the ground, the projectile skidded along the ground for a limited distance before bouncing back into the air (in some cases in excess of 2 m) retaining 64% of the impact velocity; multiple bounces were recorded. The range of distances to the rounds’ final resting positions ranged from 288 to 402 m. This suggests that the clothing system could provide some protection from ricochet threats.

### Perforating wounding potential

**Fig. 6:**
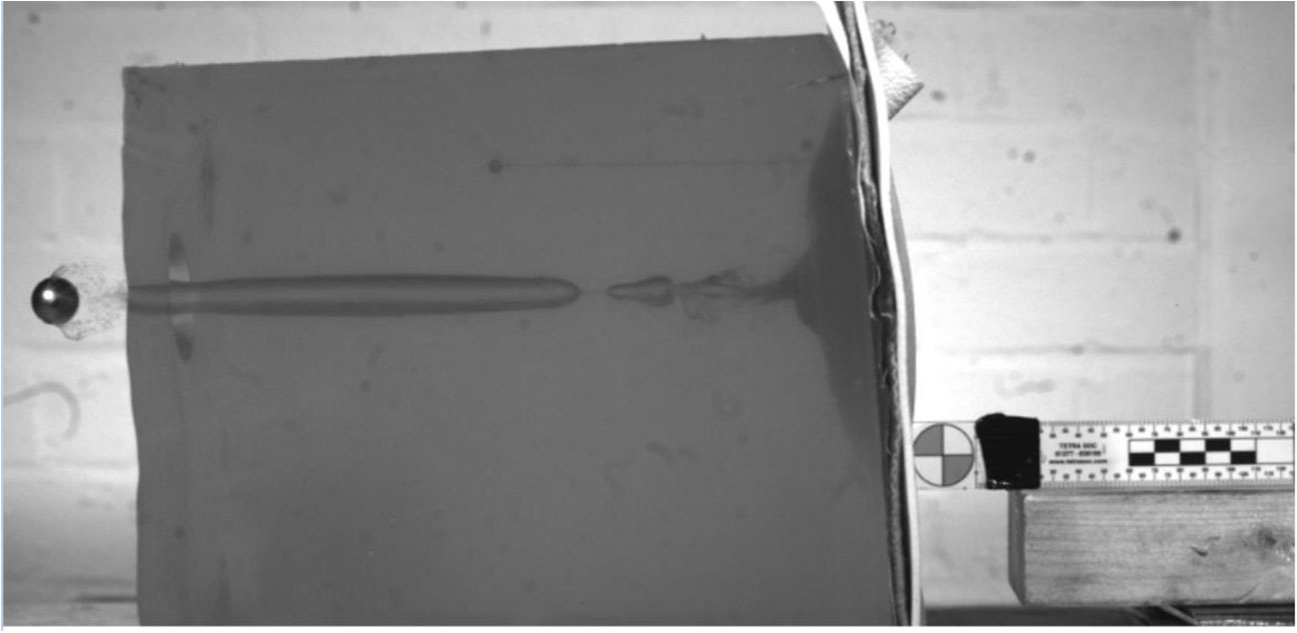
Typical example of a projectile perforating the gelatine

Shots that perforated the clothing system also perforated the gelatine block (Fig. 6). Human anthropometric data from 1981 for the British male thorax (19 to 45 years) suggests a 50th percentile chest depth of 240 mm [22]. Therefore, the perforating shots were likely to significantly penetrate the thorax of the wearer of the clothing system, although the target contained no bony structures.

**Fig. 7:**
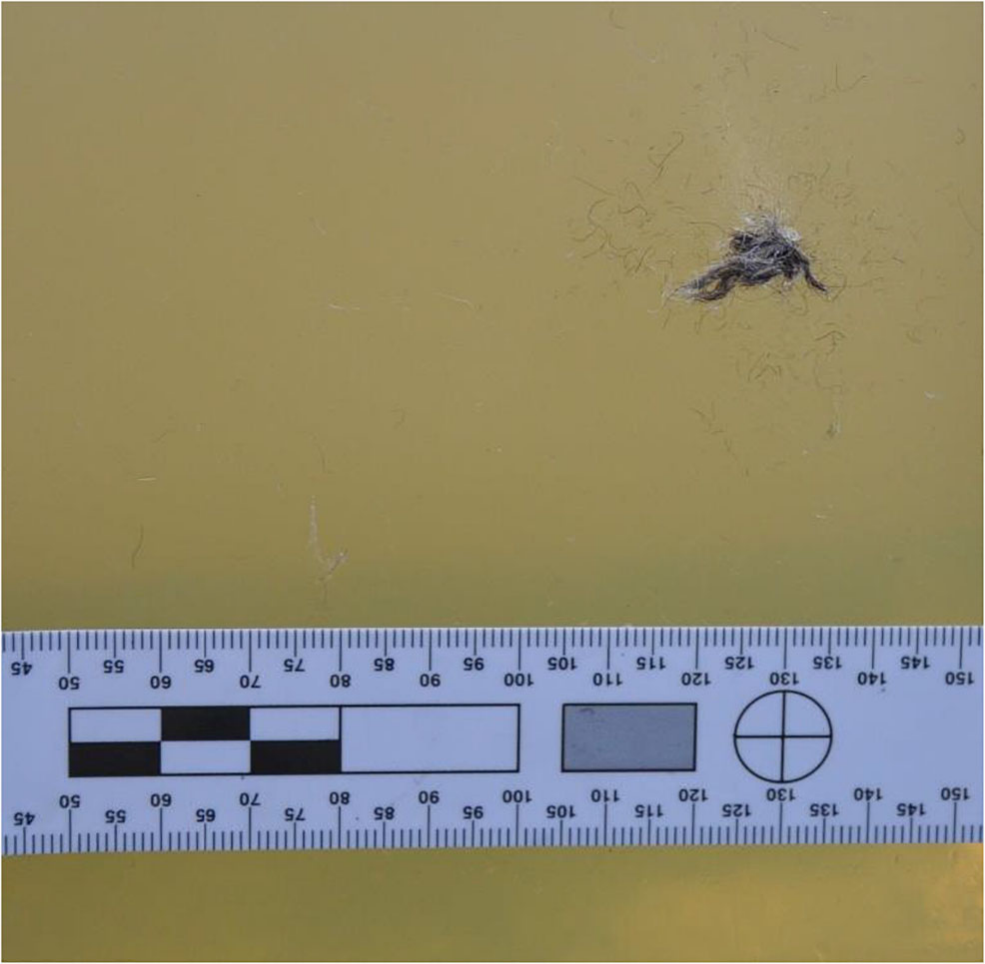
Typical example of textile debris deposited in the gelatine due to a penetrating shot

Wiseman, writing 1705, commented that ‘wounds made by gun-shot are the most complicate sort of wounds that can be inflicted’ ([25], p. 385). He also commented ‘…for the Bullet pierceth not any Part without carrying Rags along with it, which corrupt in the Wound and make Apostemations, occasioning a prolonging the Cure…Nay, while any of the Rags remain in the Wound, it will never cure ([25], p. 387).’ Evidence of clothing layer fabric debris inside the wound tract was observed during the experiments conducted (Fig. 7) and has been reported in many modern wound ballistic studies. Such debris typically requires removal increasing the level of surgical intervention.

### Non-perforating wounding potential

Behind armour blunt trauma (BABT) is ‘…the non-penetrating injury resulting from the rapid deformation of armours covering the body’ [8]. Test standards for modern body armour are designed to prevent serious BABT injuries; bruising and damage to the ribs are relatively common [9]. Such injuries were familiar to Wiseman who wrote:

And this hath happened to many in Service, who have been brought to me as mortally wounded, whereas upon search, I have found the impression only upon the greasy leather Jerkin, or their bellies black and a little scratch by the bullet. Yet these are even subject to Tumour…In others the Skin, and the Flesh under it, is sometimes wounded, and no farther. These are by us called Wounds of the Belly, not penetrating and are cured as Gun-shot Wounds in Fleshy parts...For the bullet is for the most part carried with such force, that it not only wounds the fleshy parts but also pierces the Peritoneum, hurting most an end the Internals; it being indeed impossible that the Bullet piercing the Parts containing should miss the parts contained, which are soft and tender…I have seen sometimes in the Wars a Soldier shot scarce to the Peritoneum yet the Contusion hath been so great, that the Peritoneum hath come off upon Digestion: In which case the Bowels commonly suffer under severe Colicks, and there ariseth Difficulty of breathing ([25], p. 408).

**Fig. 8:**
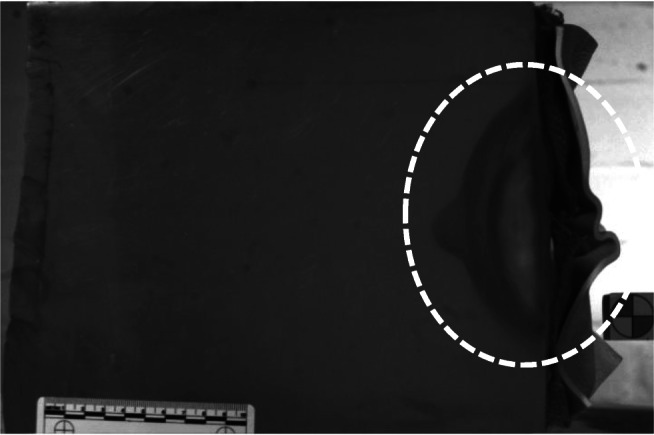
Maximum deformation into a gelatine block of a non-perforating impact (highlighted)

Figure 8 is extracted from a high-speed video of a non-perforating impact of a 12-bore lead sphere impacting the clothing layers mounted on a 10% (by mass) gelatine block at 111 m/s and shows the deformation behind the clothing layers into the gelatine. The depth of the deformation is known as the back-face signature (BFS) and in the four non-perforating impacts recorded for the 12-bore lead spheres were 50 mm, 54 mm, 60 mm and 62 mm. Similar images and BFS data have been reported for modern body armour mounted on gelatine blocks [17]. The armour packs tested by Malbon et al. [17] were to the approved standard for police body armour in the UK, suggesting that the 12-bore impacts would have led to injuries similar to those seen in the modern scenario, i.e. bruises and damaged ribs as reported by Wiseman [25].

**Fig. 9:**
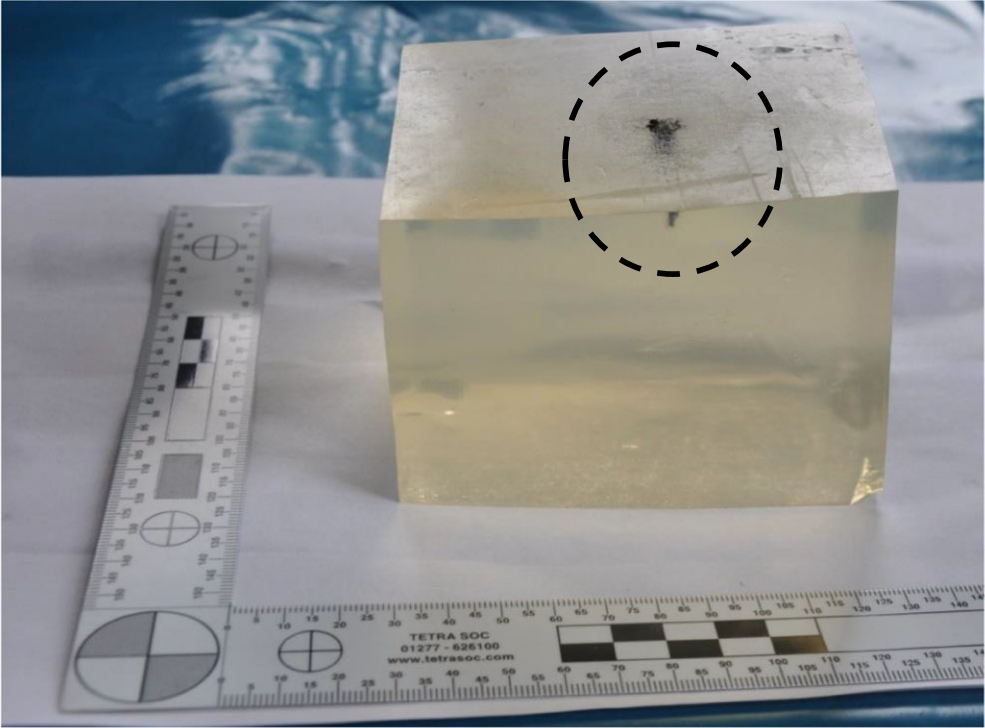
Pencilling wound pattern from non-penetrating shot highlighted by ink (highlighted)

**Fig. 10:**
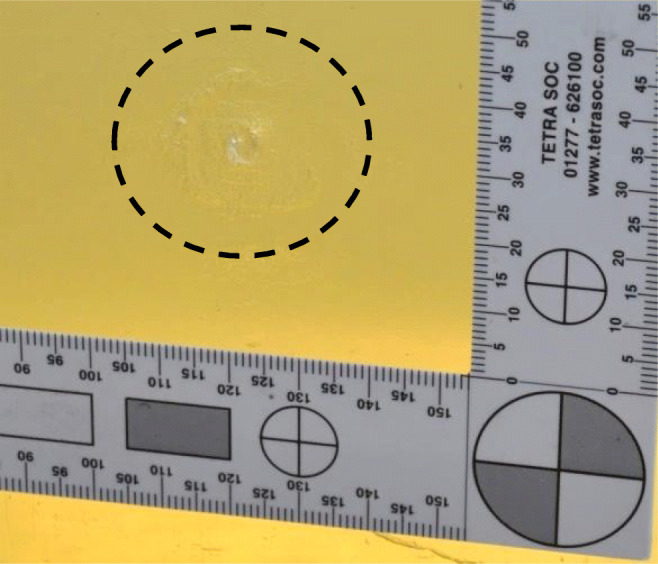
Non-penetrating shot showing the image of the muslin material impressed into the gelatine material (highlighted)

Examination of the clothing layers identified permanent deformation that matched the diameter of the 12-bore lead sphere (Fig. 9). Transfer of the linen weave pattern to the gelatine block was also noted over the diameter of the elastic deformation observed in Fig. 10.

Permanent damage of the gelatine block was noted for the non-perforating impacts. This damage was approximately 4-mm wide and 15-mm deep channel; this can be considered to be an example of pencilling which is defined as ‘a narrow indentation of soft body armour into the ballistic backing material in instances where the armour has not been perforated’ (Reference 2017 Body armour standard on gov.uk). This is similar to Wiseman’s description of wounds where ‘the Skin, and the Flesh under it, is sometimes wounded, and no farther’ [25]. Pencilling is of interest in modern body armour testing and if observed is specifically reported.

## Conclusions

One type of clothing system, more common amongst cavalrymen than infantrymen, was the linen shirt, wool waistcoat and buff-coat. The ballistic protective performance (*V*_50_) of this clothing system mounted on a tissue simulant was estimated as 102 m/s for the projectiles used. However, it should be noted that the modern projectiles were harder than historical projectiles; therefore, it is likely that the clothing system would have provided a greater level of protection from historical projectiles. The distance which the 12-bore lead projectile would travel from the end of the weapon’s muzzle to reach this velocity was calculated as 607 m/s. Given engagement distances could be as short as 5 m, it is unlikely that the clothing system would have provided protection from a direct hit. However, when considering published ricochet data and historical accounts, it is possible that the wearer would have been protected by the clothing system. Both non-perforating and perforating shots resulted in damage to the tissue simulant commensurate with modern hand-gun injuries and historical accounts.
